# Supplementary material for: Sex-Specific Outcomes in Patients with Acute Coronary Syndrome
Source: J Clin Med. 2020 Jul 6;9(7):2124. doi: 10.3390/jcm9072124 (PMC7408894; doi:10.3390/jcm9072124)
Supplement: Supplementary file 1 [file jcm-09-02124-s001.pdf]

Supplementary Material

Table S1. ICD and OPS codes used for the analyses.

	ICD/OPS code
ACS	I20.0, I21, I22
UAP	I20.0
MI	I21, I22
STEMI	I21.0-21.3
NSTEMI	I21.4
Hypertension	I10
Dyslipidemia	E78
Diabetes	E10, E11
PAD	I70.2
Prior stroke	I63, I64
Atrial fibrillation	I48
COPD	J44
Renal insufficiency	N18, N19
Prior CABG	Z95.1
Coronary angiography	OPS 1-275
In-hospital PCI	OPS 8-837
In-hospital CABG	OPS 5-361, 5-362

Table S2. Characteristics for females and males according to type of stenting comparing bare-metal-stents (BMS) and drug-eluting-stents (DES).

	Females		Males	
	BMS	DES	BMS	DES
N available	199,795	229,814	456,149	580,244
Age. years	72.2 ± 11.4	69.9 ± 11.7	65.3 ± 12.2	64.3 ± 12.0
Hypertension	120,277 (60.2%)	143,713 (62.5%)	258,315 (56.6%)	350,230 (60.4%)
Dyslipidemia	92,996 (46.6%)	119,871 (52.2%)	226,044 (49.6%)	319,638 (55.1%)
Diabetes	56,048 (28.1%)	74,234 (32.3%)	98,054 (21.5%)	152,206 (26.2%)
PAD	7368 (3.7%)	10,099 (4.4%)	19,151 (4.2%)	26,962 (4.7%)
Prior Stroke	1862 (0.9%)	1723 (0.8%)	2827 (0.6%)	2723 (0.5%)
Atrial Fibrillation	34,258 (17.2%)	28,883 (12.6%)	60,840 (13.3%)	59,869 (10.3%)
COPD	11,019 (5.5%)	12,972 (5.6%)	27,035 (5.9%)	30,637 (5.3%)
Renal Insufficiency	36,715 (18.4%)	44,148 (19.2%)	67,409 (14.8%)	86,905 (15.0%)

Prior CABG	5,904 (3.0%)	8637 (3.8%)	22,277 (4.9%)	34,392 (5.9%)
During Hospital Stay				
Mean Hospital Stay Duration, days	8.0 ± 7.6	7.2 ± 6.8	7.2 ± 7.7	6.4 ± 6.2
Coronary Angiography	194,875 (97.5%)	224,763 (97.8%)	444,679 (97.5%)	567,598 (97.8%)
In-hospital PCI	199,795 (100%)	229,814 (100%)	456,149 (100%)	580,244 (100%)
In-hospital CABG	812 (0.4%)	642 (0.3%)	2348 (0.5%)	1825 (0.3%)
In-hospital Mortality	12,457 (6.2%)	9359 (4.1%)	20,188 (4.4%)	17,001 (2.9%)

Table S3. Characteristics of **female** patients with ACS displayed per year.

	**2005**	**2006**	**2007**	**2008**	**2009**
N available	140,755	134,914	135,270	128,374	122,340
Age. years	72.7 ± 11.9	72.8 ± 12,0	72.9 ± 12.1	73.1 ± 12.0	73.2 ± 12.2
Hypertension	80,346 (57.1%)	77,540 (57.5%)	76,326 (56.4%)	72,093 (56.2%)	70,258 (57.4%)
Dyslipidemia	50,992 (36.2%)	49,555 (36.7%)	49,705 (36.7%)	47,882 (37.3%)	47,305 (38.7%)
Diabetes	40,858 (29.0%)	39,415 (29.2%)	40,370 (29.8%)	38,265 (29.8%)	36,354 (29.7%)
PAD	4757 (3.4%)	4125 (3.1%)	4641 (3.4%)	4876 (3.8%)	4583 (3.7%)
Prior Stroke	1656 (1.2%)	1511 (1.2%)	1473 (1.1%)	1337 (1.0%)	1172 (1.0%)
Atrial Fibrillation	24,353 (17.3%)	23,745 (17.6%)	24,493 (18.1%)	23,756 (18.5%)	23,305 (19.0%)
COPD	8376 (6.0%)	7968 (5.9%)	8138 (6.0%)	7889 (6.1%)	7873 (6.4%)
Renal Insufficiency	21,728 (15.4%)	21,922 (16.2%)	23,845 (17.6%)	24,824 (19.3%)	25,824 (21.1%)
Prior CABG	5845 (4.2%)	5875 (4.4%)	5957 (4.4%)	5771 (4.5%)	5669 (4.6%)
During Hospital Stay					
Mean Hospital Stay Duration, days	8.4 ± 8.0	8.3 ± 8.0	8.1 ± 8,0	7.9 ± 7.8	7.7 ± 7.9
Coronary Angiography	63,248 (44.9%)	63,910 (47.3%)	67,088 (49.6%)	66,191 (51.6%)	66,770 (54.6%)
In-hospital PCI	37,193 (26.4%)	38,757 (28.7%)	40,733 (30.1%)	41,441 (32.3%)	40,745 (33.3%)
In-hospital CABG	4588 (3.3%)	4440 (3.3%)	4302 (3.2%)	4057 (3.2%)	3823 (3.1%)
In-hospital Mortality	11,582 (8.2%)	11,056 (8.2%)	11,217 (8.3%)	10,815 (8.4%)	10,332 (8.4%)

	**2010**	**2011**	**2012**	**2013**	**2014**	**2015**
N available	123,051	120,722	120,803	116,524	115,000	108,292
Age. years	73.1 ± 12.3	73.4 ± 12.2	73.2 ± 12.4	73.3 ± 12.4	73.3 ± 12.4	73.2 ± 12.4
Hypertension	71,083 (57.8%)	70,740 (58.6%)	71,061 (58.8%)	68,430 (58.7%)	67,469 (58.7%)	63,780 (58.9%)
Dyslipidemia	49,214 (40.0%)	49,243 (40.8%)	50,018 (41.4%)	47,861 (41.1%)	47,742 (41.5%)	45,576 (42.1%)
Diabetes	36,908 (30.0%)	36,813 (30.5%)	36,137 (29.9%)	35,083 (30.1%)	34,202 (29.7%)	32,068 (29.6%)
PAD	4922 (4.0%)	4962 (4.1%)	5034 (4.2%)	5109 (4.4%)	5352 (4.7%)	4929 (4.6%)
Prior Stroke	1136 (0.9%)	1039 (0.9%)	1118 (0.9%)	1064 (0.9%)	1029 (0.9%)	964 (0.9%)
Atrial Fibrillation	24,043 (19.5%)	24,636 (20.4%)	24,764 (20.5%)	24,228 (20.8%)	24,231 (21.1%)	22,832 (21.1%)

COPD	8198 (6.7%)	8323 (6.9%)	8483 (7.0%)	8459 (7.3%)	8344 (7.3%)	8087 (7.5%)
Renal Insufficiency	26,013 (21.1%)	25,855 (21.4%)	26,668 (22.1%)	27,225 (23.4%)	27,730 (24.1%)	25,858 (23.9%)
Prior CABG	6094 (5.0%)	5736 (4.8%)	5810 (4.8%)	5551 (4.8%)	5532 (4.8%)	5186 (4.8%)
During Hospital Stay						
Mean Hospital Stay Duration, days	7.5 ± 7.7	7.5 ± 7.7	7.4 ± 7.8	7.2 ± 7.5	7.1 ± 7.4	6.8 ± 7.0
Coronary Angiography	70,234 (57.1%)	70,727 (58.6%)	72,111 (59.7%)	71,491 (61.4%)	72,843 (63.3%)	69,879 (64.6%)
In-hospital PCI	43,295 (35.2%)	44,782 (37.1%)	45,677 (37.8%)	45,959 (39.4%)	47,563 (41.4%)	46,276 (42.7%)
In-hospital CABG	3902 (3.2%)	3743 (3.1%)	3824 (3.2%)	3816 (3.3%)	3577 (3.1%)	3336 (3.1%)
In-hospital Mortality	9725 (7.9%)	9201 (7.6%)	9007 (7.5%)	8832 (7.6%)	8114 (7.1%)	7766 (7.2%)

Table S4. Characteristics of **male** patients with ACS displayed per year.

	2005	2006	2007	2008	2009
N available	232,444	225,849	229,699	221,053	215,025
Age. years	65.6 ± 12.2	65.7 ± 12.4	66.0 ± 12.4	66.3 ± 12.4	66.4 ± 12.5
Hypertension	130,153 (56.0%)	126,688 (56.1%)	127,636 (55.6%)	122,561 (55.4%)	121,724 (56.6%)
Dyslipidemia	100,662 (43.3%)	97,217 (43.1%)	99,627 (43.4%)	97,247 (44.0%)	97,218 (45.2%)
Diabetes	53,755 (23.1%)	53,571 (23.7%)	56,007 (24.4%)	55,083 (24.9%)	54,973 (25.6%)
PAD	11,218 (4.8%)	9612 (4.3%)	10,818 (4.7%)	11,434 (5.2%)	11,412 (5.3%)
Prior Stroke	1987 (0.9%)	1823 (0.8%)	1808 (0.8%)	1566 (0.7%)	11,217 (5.2%)
Atrial Fibrillation	30,371 (13.1%)	30,267 (13.4%)	31,872 (13.9%)	31,878 (14.4%)	31,988 (14.9%)
COPD	16,840 (7.2%)	16,377 (7.3%)	16,190 (7.0%)	15,784 (7.1%)	15,977 (7.4%)
Renal Insufficiency	33,080 (14.2%)	33,776 (15.0%)	36,393 (15.8%)	37,539 (17.0%)	39,443 (18.3%)
Prior CABG	18,183 (7.8%)	17,783 (7.9%)	18,056 (7.9%)	17,917 (8.1%)	17,903 (8.3%)
During Hospital Stay					
Mean Hospital Stay Duration, days	7.4 ± 7.5	7.3 ± 7.6	7.2 ± 7.7	7.2 ± 7.7	7.0 ± 7.7
Coronary Angiography	132,468 (57.0%)	133,363 (59.1%)	140,254 (61.1%)	138,469 (62.6%)	141,169 (65.7%)
In-hospital PCI	89,074 (38.3%)	92,162 (40.8%)	97,023 (42.2%)	98,170 (44.4%)	97,932 (45.5%)
In-hospital CABG	13,115 (5.6%)	12,728 (5.6%)	12,745 (5.5%)	12,323 (5.6%)	12,183 (5.7%)
In-hospital Mortality	12,450 (5.4%)	12,491 (5.5%)	12,654 (5.5%)	12,427 (5.6%)	12,506 (5.8%)

	2010	2011	2012	2013	2014	2015
N available	220,776	218,991	223,809	217,304	217,404	209,146
Age. years	66.5 ± 12.6	66.7 ± 12.6	66.7 ± 12.7	66.8 ± 12.7	66.9 ± 12.7	67.0 ± 12.7
Hypertension	127,149 (57.6%)	127,055 (58.0%)	129,519 (57.9%)	127,277 (58.6%)	126,804 (58.3%)	121,749 (58.2%)
Dyslipidemia	103,209 (46.7%)	103,693 (47.4%)	107,483 (48.0%)	103,651 (47.7%)	104,844 (48.2%)	101,255 (48.4%)
Diabetes	58,011 (26.3%)	58,480 (26.7%)	59,926 (26.8%)	58,933 (27.1%)	59,206 (27.2%)	57,241 (27.4%)
PAD	11,875 (5.4%)	12,178 (5.6%)	12,388 (5.5%)	12,387 (5.7%)	12,736 (5.9%)	12,365 (5.9%)

Prior Stroke	1547 (0.7%)	1478 (0.7%)	1454 (0.6%)	1486 (0.7%)	1601 (0.7%)	1506 (0.7%)
Atrial Fibrillation	33,916 (15.4%)	35,226 (16.1%)	37,139 (16.6%)	36,626 (16.9%)	37,670 (17.3%)	36,826 (17.6%)
COPD	16,615 (7.5%)	16,752 (7.6%)	16,764 (7.5%)	16,210 (7.5%)	16,297 (7.5%)	15,910 (7.6%)
Renal Insufficiency	39,559 (17.9%)	39,256 (17.9%)	39,970 (17.9%)	40,633 (18.7%)	41,417 (19.1%)	39,229 (18.8%)
Prior CABG	18,809 (8.5%)	18,579 (8.5%)	18,861 (8.4%)	18,087 (8.3%)	18,230 (8.4%)	17,347 (8.3%)
During Hospital Stay						
Mean Hospital Stay Duration, days	6.8 ± 7.5	6.8 ± 7.5	6.8 ± 7.6	6.7 ± 7.5	6.6 ± 7.4	6.4 ± 6.9
Coronary Angiography	148,780 (67.4%)	151,294 (69.1%)	156,223 (69.8%)	154,941 (71.3%)	157,796 (72.6%)	153,857 (73.6%)
In-hospital PCI	104,386 (47.3%)	107,026 (48.9%)	110,809 (49.5%)	111,511 (51.3%)	114,603 (52.7%)	113,369 (54.2%)
In-hospital CABG	12,426 (5.6%)	11,895 (5.4%)	12,302 (5.5%)	12,594 (5.8%)	12,483 (5.7%)	11,547 (5.5%)
In-hospital Mortality	12,112 (5.5%)	11,687 (5.3%)	11,759 (5.3%)	11,837 (5.4%)	11,324 (5.2%)	11,064 (5.3%)

Table S5. Age-Adjusted incidence rates of ACS, NSTEMI, STEMI, UAP and in-hospital mortality cases per 1000 person-years ranging from 2005 to 2015 for females and males. The incidences for ACS, NSTEMI, STEMI, and UAP were calculated by using the age-specific distribution in Germany at the 31st December of each year, while incidences of in-hospital mortality were calculated by using the ACS distribution in Germany of each year.

			Female					Male		
**Year**	**ACS**	**NSTEMI**	**STEMI**	**UAP**	**Mortality**	**ACS**	**NSTEMI**	**STEMI**	**UAP**	**Mortality**
2005	4.26	1.01	1.12	1.88	87.17	7.65	1.67	2.27	3.36	57.49
2006	4.03	1.12	1.00	1.71	84.99	7.30	1.88	2.08	3.05	58.43
2007	3.99	1.27	0.93	1.66	84.58	7.28	2.11	1.98	2.98	56.95
2008	3.74	1.30	0.85	1.48	84.53	6.87	2.20	1.80	2.70	56.94
2009	3.51	1.28	0.76	1.38	83.91	6.54	2.17	1.67	2.55	58.28
2010	3.48	1.34	0.72	1.34	78.22	6.58	2.28	1.65	2.52	54.62
2011	3.40	1.40	0.70	1.23	74.37	6.56	2.47	1.62	2.36	52.36
2012	3.38	1.43	0.68	1.20	73.22	6.60	2.58	1.59	2.32	51.27
2013	3.24	1.41	0.64	1.12	74.63	6.32	2.57	1.52	2.13	52.97
2014	3.16	1.37	0.63	1.10	69.68	6.23	2.54	1.51	2.09	50.68
2015	2.96	1.34	0.58	0.99	70.98	5.92	2.54	1.41	1.90	51.29

Table S6. Crude rates of ACS and in-hospital mortality per age categories for females and males.

	**Females**		**Males**	
**Age categories**	**ACS**	**In-hospital mortality**	**ACS**	**In-hospital mortality**
18 to < 30	1,582 (0.12 %)	38 (2.4 %)	5,560 (0.23 %)	68 (1.22 %)
≥ 30 to < 40	9120 (0.67 %)	199 (2.18 %)	36,334 (1.49 %)	602 (1.66 %)
≥ 40 to < 50	56,136 (4.11 %)	1190 (2.12 %)	217,533 (8.95 %)	4182 (1.92 %)
≥ 50 to < 60	134,515 (9.85 %)	3074 (2.29 %)	464,968 (19.12 %)	11,963 (2.57 %)
≥ 60 to < 70	246,496 (18.04 %)	8459 (3.43 %)	608,440 (25.02 %)	23,889 (3.93 %)

≥ 70 to < 80	454,894 (33.3 %)	27,247 (5.99 %)	732,886 (30.14 %)	45,922 (6.27 %)
≥ 80 to < 85	231,320 (16.93 %)	24,991 (10.8 %)	228,421 (9.39 %)	23,328 (10.21 %)
≥ 85 to < 90	158,252 (11.58 %)	24,966 (15.78 %)	105,143 (4.32 %)	15,207 (14.46 %)
≥ 90	72,653 (5.32 %)	17,129 (23.58 %)	31,768 (1.31 %)	7040 (22.16 %)
